# Sputtering of silicon nanopowders by an argon cluster ion beam

**DOI:** 10.3762/bjnano.10.13

**Published:** 2019-01-10

**Authors:** Xiaomei Zeng, Vasiliy Pelenovich, Zhenguo Wang, Wenbin Zuo, Sergey Belykh, Alexander Tolstogouzov, Dejun Fu, Xiangheng Xiao

**Affiliations:** 1 Department of Physics and Key Laboratory of Artificial Micro- and Nano-structures of Ministry of Education, Hubei Nuclear Solid Physics Key Laboratory and Center for Ion Beam Application, School of Physics and Technology, Wuhan University, Wuhan, 430072, China; 2 School of Power & Mechanical Engineering, Wuhan University, Wuhan, 430072, China; 3 Ryazan State Radio Engineering University, Gagarin Str. 59/1, Ryazan, 390005, Russian Federation; 4 Centre for Physics and Technological Research (CeFITec), Dept. de Física da Faculdade de Ciências e Tecnologia (FCT), Universidade Nova de Lisboa, Caparica, 2829-516, Portugal

**Keywords:** finite size effect, gas cluster ion beam, silicon nanoparticles, smoothing effect, sputtering

## Abstract

In this work an Ar^+^ cluster ion beam with energy in the range of 10–70 keV and dose of 7.2 × 10^14^–2.3 × 10^16^ cluster/cm^2^ was used to irradiate pressed Si nanopowder targets consisting of particles with a mean diameter of 60 nm. The influence of the target density and the cluster ion beam parameters (energy and dose) on the sputtering depth and sputtering yield was studied. The sputtering yield was found to decrease with increasing dose and target density. The energy dependence demonstrated an unusual non-monotonic behavior. At 17.3 keV a maximum of the sputtering yield was observed, which was more than forty times higher than that of the bulk Si. The surface roughness at low energy demonstrates a similar energy dependence with a maximum near 17 keV. The dose and energy dependence of the sputtering yield was explained by the competition of the finite size effect and the effect of debris formation.

## Introduction

Etching using gas clusters is generally recognized an effective technique to sputter solid materials [1]. Sputtering using gas cluster ions differs drastically from sputtering using monomer ions; this difference arises from the peculiar features of the cluster beam. Due to the large quantity of monomers which constitute each cluster (hundreds or even thousands), the kinetic energy per monomer in the cluster is only a few eV. Therefore, individual monomers cannot penetrate deeply into targets. On the other hand, the interaction between the monomers forming the cluster and the atoms of the target is highly nonlinear in comparison with the Sigmund’s collision theory [2]. Such nonlinearity and low individual kinetic energy result in dissipation of the cluster energy mostly in the near-surface region and formation of craters on the surface. The crater formation is accompanied by a huge increase in temperature and pressure in the impact area [3], resulting in ejection of a large amount of material. Effective sputtering during the cluster bombardment is also explained by the high probability of the crater formation as compared with the monomer bombardment [4]. The angular distribution of the sputtered atoms demonstrates an under-cosine shape, in other words, the atoms are sputtered mostly in the lateral direction [5]. This effect results in another prominent phenomenon called surface smoothing. Using molecular dynamics (MD) and Monte Carlo simulations it has been shown that the effect of the cluster impact depends on the surface morphology, with preferable erosion of hills as compared with valleys [6], which also results in the smoothing of the surface [7].

Sputtering by an argon cluster beam has been studied for many pure metals (Cu, Ag, Au, W, Pt, Ni) and their alloys, semiconductors (Si and SiC), and insulators (SiO_2_ and diamond) [1,8–12]. In comparison with bombardment by a monomer beam, cluster irradiation increases the sputtering yield *Y* (estimated as a number of the sputtered target atoms per cluster ion) by one order of magnitude. No remarkable dose effect has been observed, i.e., the sputtering depth shows linear dependence on the cluster ion dose [8,12–13]. The energy dependence of the sputtering yield of Ag, Cu, Au, and Si exhibits a linear or ultralinear behavior [8,14–15]. In both cases, the energy threshold of 3–6 keV is observed. This value correlates with the surface binding energy of the target materials [14].

All of the above-mentioned sputtering experiments have been carried out for massive poly- or single-crystalline solid targets under bombardment by cluster-like projectiles. However, sputtering effects also can be enhanced in finite size systems such as nanoparticles or nanowires. There are many molecular dynamics simulations using the collision cascade theory and, at the same time, only a few experimental studies on the interaction of monomer and cluster projectiles with nanodimensional systems. Using a MD simulation, Kissel et al. [16] have studied the effect of the bombardment of gold nanoparticles with radius *R* = 4 nm by 100 keV gold atoms. The sputter yields ranged from only a few sputtered atoms to complete particle disintegration. Järvi et al. [17–18] have shown that the highest sputtering yield of gold nanoparticles by 25 keV gallium or 200 keV argon beams is observed for particles of ≈8 nm in diameter. The sputtering yield was about three times higher than that of bulk gold. Zimmermann et al. [19] have revealed that the sputtering yield of Au nanoparticles with a radius of 10 nm bombarded by 16 keV or 64 keV Au projectiles is more than double the number compared to the sputtering of the bulk target. The sputtering yield versus radius of a-Si or SiGe particles scaled to the energy deposition depth has been studied by Nietiadi et al. using MD and Monte Carlo simulations [20–21]. As projectiles, Ar atoms were used with an energy of 20 keV. The sputtering yield was maximium when the radius of the particle was close to the energy deposition depth. The sputtering yield of Au particles bombarded by 200 keV Xe atoms in the collision-spike sputtering regime has been also found to be more than three times higher in comparison with that of the bulk Au target [22].

Sputtering experiments have been performed mostly for gold nanoparticles and nanorods. Klimmer et al. [23] have studied the sputtering of gold nanoparticles with a radius of 3.6 nm on a sapphire substrate irradiated with 200 keV Ar ions. Their model predicts a strong size effect of the sputtering yield *Y* for radii below 200 nm. For particles with radii less than 25 nm, a parabolic dependence *Y*(*R*) = *kR*^2^ is predicted, where *k* is a constant and *R* is the radius of the nanoparticle. The sputtering yield within the range of 100–1900 atoms/ion (compared with values for a flat surface of 50) has been revealed for 80 keV Xe ion irradiation of Au nanowires of 20 nm in diameter and micrometers in length [24–25]. The sputtering yield dependence on the gold particle size under bombardment with 20 keV С_60_ ions has been studied in [26]. At 9.3 nm the particle diameter the yield was 320, whereas for 98.8 nm particles and thin films, the yield was 96 and 129, respectively.

In this study, we combine two of the above-mentioned approaches to further increase the sputtering yield, i.e., employing heavy cluster projectiles to irradiate finite size targets. The feasibility of such an increase in the sputtering yield has already been discussed by Belykh et al. [27]. In the present work, we use massive argon clusters, as projectiles, and pressed Si nanopowder consisting of nanoparticles with a mean particle diameter of 60 nm, as the sputtered material. The sputtering yield dependence versus average target density, cluster dose and cluster energy are studied and compared with the data for the bulk Si target.

## Experimental

To produce the argon cluster ion beam we use a custom-built gas cluster accelerator described elsewhere [28]. In this study, a conical metal nozzle with a throat diameter of 65 µm is used, the pressure in the gas source is *P* = 10 bar, and the ionizing electron energy is 150 eV. The time and area averaged cluster current *I* in µA on the 5 × 5 mm^2^ target depends on the accelerating voltage *U* in kV and can be described by an empirical equation: *I* = –0.007*U*^2^ + 0.2*U* – 0.53. The gas pressure at the full gas supply is 0.13 Pa in the nozzle chamber, 5 × 10^−3^ Pa in the ionization chamber, and 3 × 10^−3^ Pa in the processing chamber. The base gas pressure is 2 × 10^−4^ Pa.

**Figure 1:**
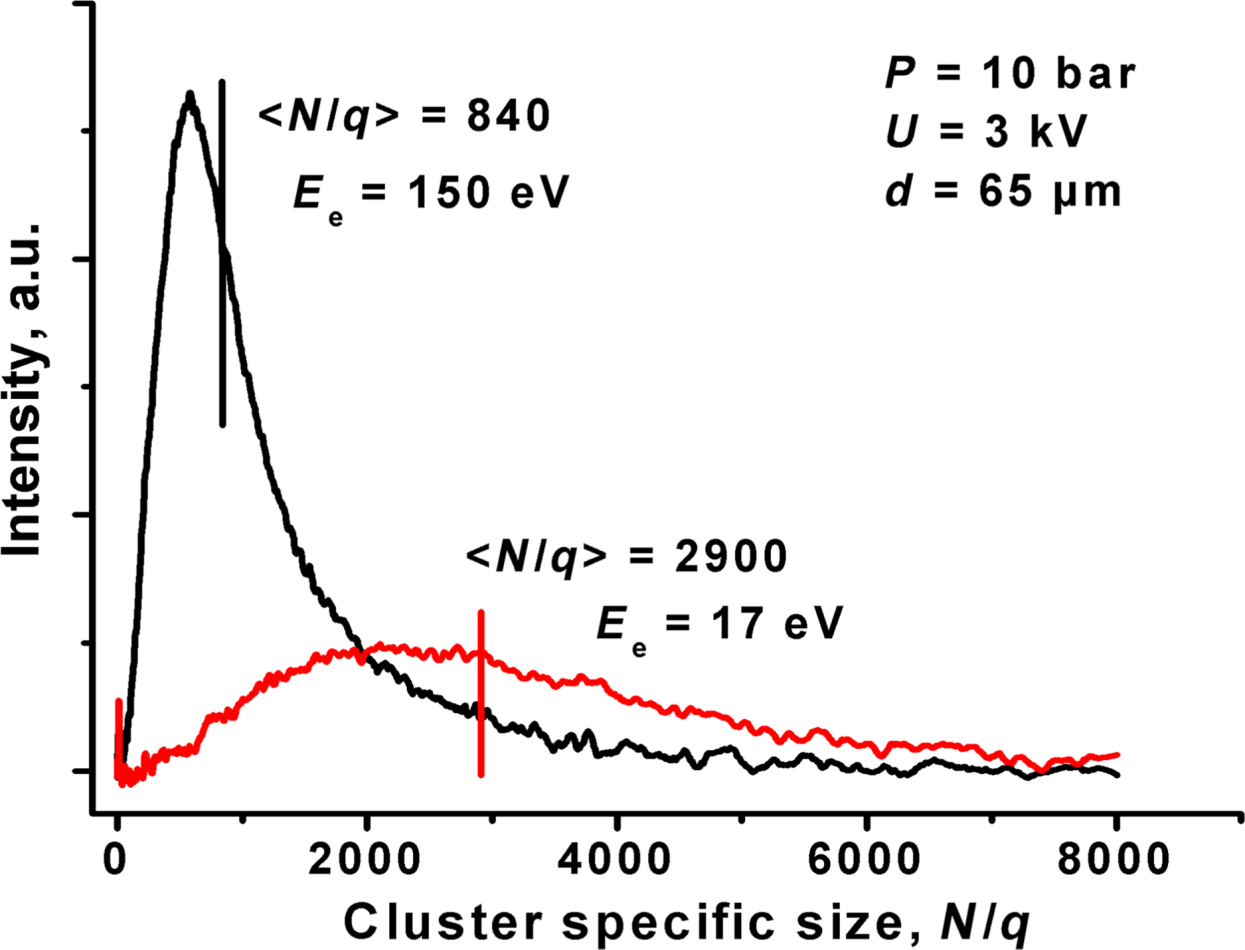
Mass spectra of the argon cluster beam ionized by electrons with the energies of 17 and 150 eV. The vertical lines represent the mean cluster specific sizes 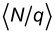.

In Figure 1 two time-of-flight mass spectra of the cluster beam ionized by an electron beam with different energies *E*_e_ are shown. The electron energy influences the mass spectra rather profoundly. The mean cluster specific sizes 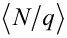 are 2900 and 840 atoms at *E*_e_ = 17 and 150 eV, respectively. It is known that the effect of the cluster fragmentation under electron impact is negligible for massive clusters (*N* > 100) [29]. Therefore, the decrease of the mean cluster specific size at higher electron ionization energy can be interpreted as a formation of multiple ionized cluster ions [30]. The multiple ionization is also characterized by some charge distribution with the most probable value, which we designate as an effective charge, *q*_eff_. Thus, the multiple ionization results in a higher speed obtained by the cluster ions in the accelerating field and, as a consequence, in a decrease of the estimated specific size 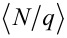 in *q*_eff_ times. Given that at low *E*_e_ = 17 eV only singly charged cluster ions are formed with


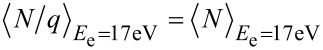


(first ionization potential of Ar is 15.8 V) and an absence of cluster defragmentation, then at *E*_e_ = 150 eV we can estimate the effective charge *q*_eff_ as


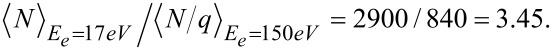


Therefore, in our experiment “mean” 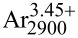 clusters are employed. The multiple ionization influences the energy of the cluster ion, i.e., the 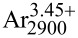 cluster in the accelerating voltage of *U* obtains an energy of 3.45·*e·U*, where *e* is the electron charge. It should also be noted that the multiple ionization complicates the ion dose measurement, namely, the actual number of the cluster ions reaching the target becomes smaller by the factor *q*_eff_ compared to the measured dose, i.e., the charge collected by the target. In this paper, we use cluster/cm^2^ as a dose unit.

For the sputtering experiments, we have prepared samples of pressed Si nanopowder with a spherical particle diameter of 60 nm (Shanghai Yao Tian New Material Co., Ltd., oxygen content <2 atom %, the particles were prepared by plasma arc techniques). The Si nanopowder was pressed into pellets with a diameter of 17 mm and thickness of 160–290 µm at different loads during 10 min by a mechanical press. Then, the pellets were cut into 5 × 5 mm^2^ samples and mounted on a steel substrate holder of the same size. Since in the pressed powder the particles do not occupy all of the space we characterize the pellets by an average density, estimated as ρ *= m*/*St*, where *m* is the pellet mass, *S* is its area, and *t* is its thickness measured by an optical microscope. The sputtering yield is estimated as *Y =* atom/cluster ion = ρ*hN*_A_/µ*D*, where *h* is the sputtering depth, *N*_A_ is Avogadro’s number, µ is the silicon molar mass, and *D* is the cluster ion dose. The sputtering depth was measured using a tungsten wire with a diameter of 15 µm as a mask. Tungsten is a convenient mask material due to its low sputtering rate [31]. Before irradiation the samples were covered by a few coils of the wire mask. During irradiation the surface under the mask was not subjected to the sputtering, hence, a step was formed between the irradiated and covered surfaces. Then, the height of the step, which is equal to the sputtering depth, was measured by an atomic force microscope (AFM). As reference samples, we use a bulk single crystalline silicon. These samples before irradiation were etched in 10% HF solution to remove a surficial thin oxide layer. Both sets of the samples were irradiated with the cluster beam at a right angle to the plane of the surface, with energy in the range of 10.4–69 keV and dose of 7.2 × 10^14^–2.3 × 10^16^ cluster/cm^2^ at room temperature.

The sputtering depth and surface roughness *R*_RMS_ (root mean squared roughness) were monitored by AFM with a Shimadzu SPM-9500 J3 device, operated in tapping mode with a measuring area of 7 × 40 µm^2^ and 10 × 10 µm^2^, respectively. Each sputtering depth was calculated as a mean of nine values measured at different sample spots to eliminate any possible nonuniformity of the cluster beam.

## Results and Discussion

### Average density dependence

**Figure 2:**
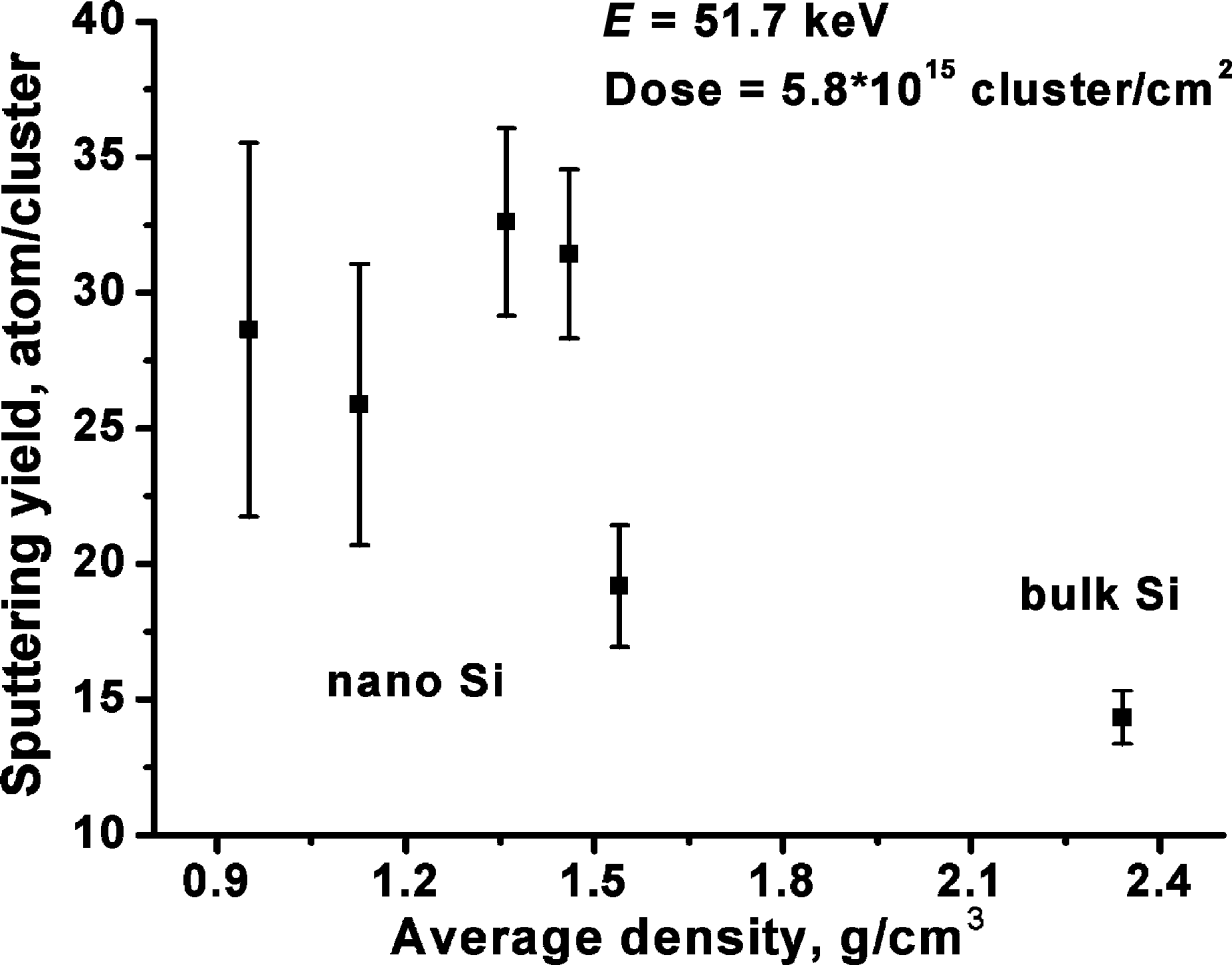
Sputtering yield versus the average density of the pressed nanopowder. Data on bulk Si is also shown. The cluster ion energy is 51.7 keV and the ion dose is 5.8 × 10^15^ cluster/cm^2^.

Figure 2 shows the sputtering yield versus the average density of the pressed nanopowder and bulk Si samples bombarded by the cluster beam with the ion dose of 5.8 × 10^15^ cluster/cm^2^ at an energy of 51.7 keV. The average density ranged from 0.95–1.54 g/cm^3^. One can see that the sputtering yield increases from 14.3 to 28.6 atoms/cluster with decreasing average density from 2.34 g/cm^3^ (bulk Si) to 0.95 g/cm^3^, respectively. The sputtering yield reaches a maximum of 32.4 atoms/cluster at the density of 1.4 g/cm^3^. However, due to the scattering of experimental data the presence of the maximum is not clear enough. These uncertainties at low densities resulted from a huge increase of the surface roughness after the bombardment, from an initial roughness *R*_RMS_ = 6.7 nm up to a few hundreds of nanometers, which complicates the depth measurement by AFM.

We explain the increase of the sputtering yield of the nanopowder sample, in comparison with the bulk Si, by the presence of the finite size effect in such system [27], i.e., the energy of the cluster ion after impact with a certain silicon particle cannot be spread effectively at a large range inside the material due to too little contact of the particle with neighbors. This concept is proven by the increase in the sputtering yield with a decrease in average density, since the average distance between Si nanoparticles in less dense material is larger. This causes less effective energy transfer to the neighboring nanoparticles, i.e., increase in the energy density in the impact area. Particular mechanisms of the sputtering can include desorption of intact silicon nanoparticles similar to the process described in [32] as well as more or less complete disintegration of the silicon particles during collision with the cluster ions, analogous to disintegration by atomic bombardment [16]. Below we describe the results for the samples with an average density of 1.4 g/cm^3^ showing high sputtering yield.

### Dose and energy dependence

**Figure 3:**
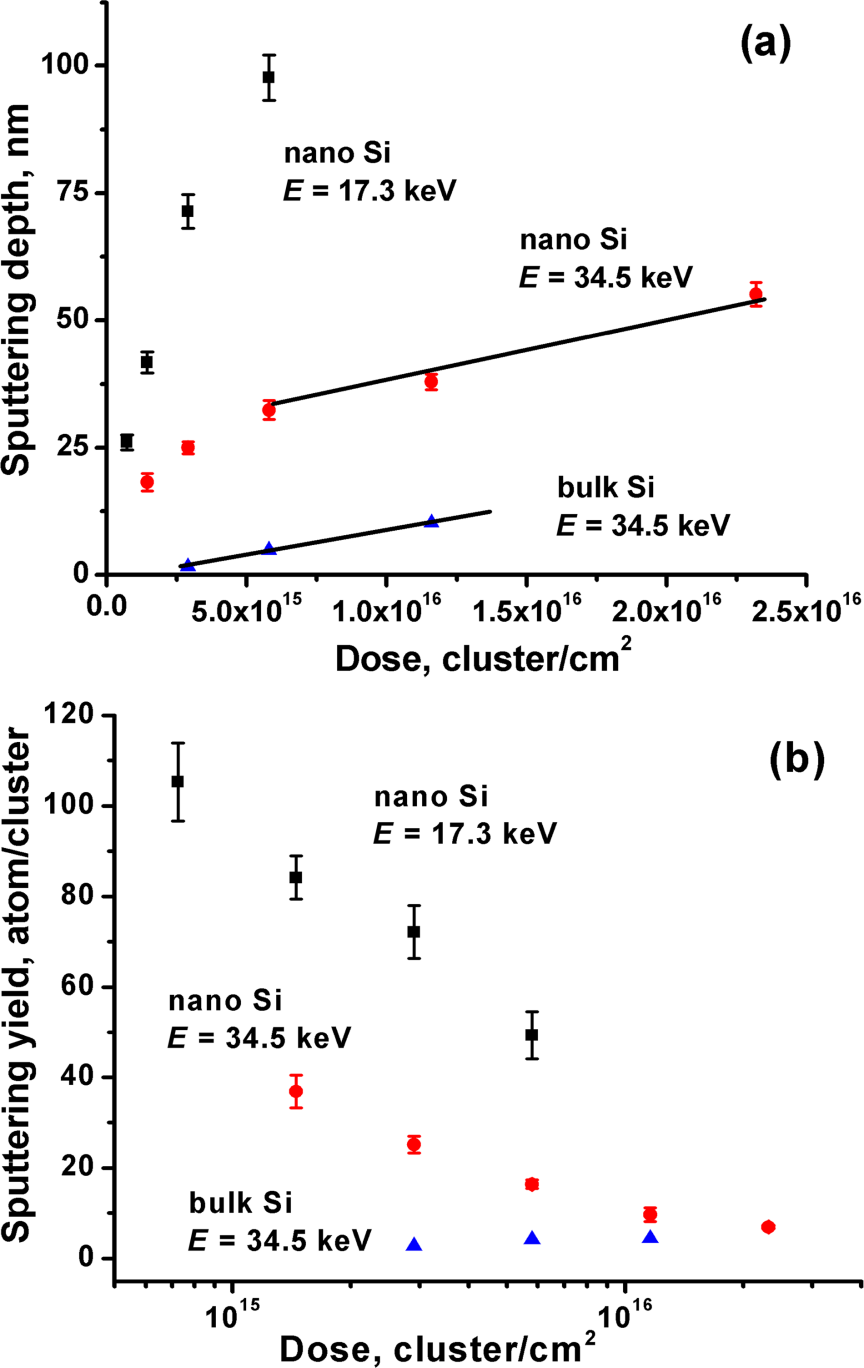
The sputtering depth (a) and sputtering yield (b) dependence on the ion dose of the nanopowder Si irradiated at different energies and bulk Si irradiated at 34.5 keV.

The dose dependence of the sputtering depth and sputtering yield of the nanopowder and bulk Si are shown in Figure 3. The sputtering depth of the bulk Si exhibits a linear dependence (Figure 3a), which suggests a nearly constant sputtering yield of 4.1 atoms/cluster (Figure 3b). This value is much lower than the sputtering yields of ≈125 and ≈85 atoms/cluster found by Ichiki et al. [15] and Seki et al. [33], respectively, at the same energy and comparable cluster specific sizes. However, in the referred studies, the authors do not define the charge state of the cluster ions, and for that reason, their actual energy is unknown. Moreover, the higher ionizing electron energy of 300 and 400 eV used in [15] and [33], respectively, causes the formation of a larger amount of multiply charged cluster ions than in the present work, which further increases the cluster ion energy and, as a consequence, the sputtering yield.

The sputtering depth and sputtering yield of the nanopowder sample irradiated at 17.3 keV are almost three times higher than those obtained at 34.5 keV and more than twenty times higher than those obtained for the bulk Si. The nanopowder samples demonstrate a steep sublinear increase of the sputtering depth at low doses with a subsequent change to a linear dependence (see the data for 34.5 keV irradiation shown in Figure 3a). The sputtering yield dependence of the nanopowder samples show a decrease with an increase in the ion dose (Figure 3b). The slope of the linear part of the sputtering depth dependence at 34.5 keV is rather close to that obtained for the bulk Si (Figure 3a). In the sputtering yield dependence, this fact is observed as neighbor values of the sputtering yields (see a region of high doses, Figure 3b).

**Figure 4:**
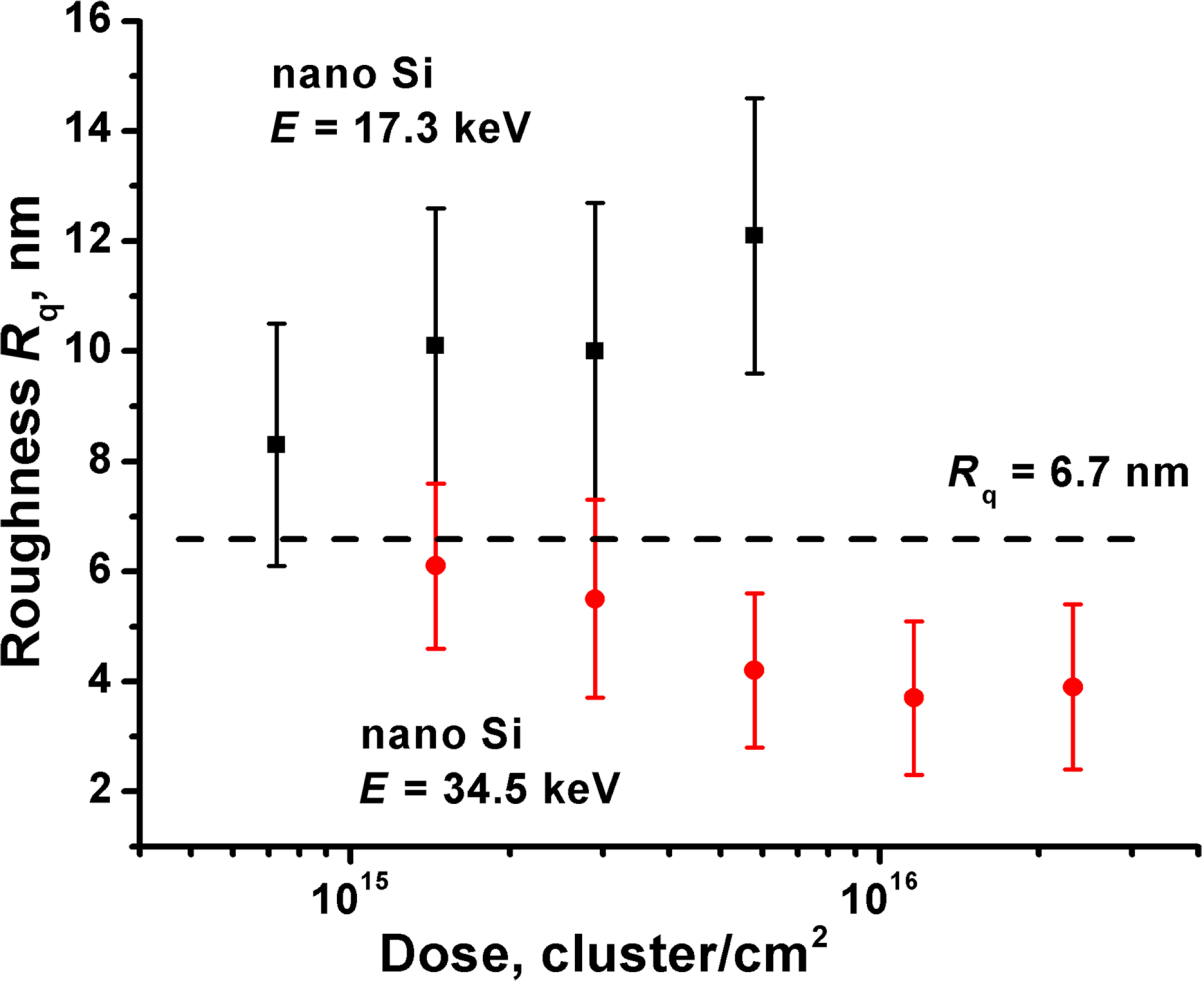
The roughness of the Si nanopowder versus the ion dose at different energies. The dashed line represents the initial roughness of the pressed pellet.

The roughness of the pressed nanopowder pellets irradiated at different doses and energies is shown in Figure 4. For the samples irradiated at 34.5 keV the smoothing effect is observed with a roughness value decreasing from initial 6.7 to final 3.9 nm at a dose of 2.3 × 10^16^ cluster/cm^2^. Whereas the samples irradiated at 17.3 keV, on the contrary, demonstrate a roughening effect. Due to the large experimental uncertainty, the dependence of the roughness versus dose is not clearly observed.

To explain the decreasing dose dependence of the sputtering yield, we propose that during the sputtering process the formation of particle debris on the surface occurs.

**Figure 5:**
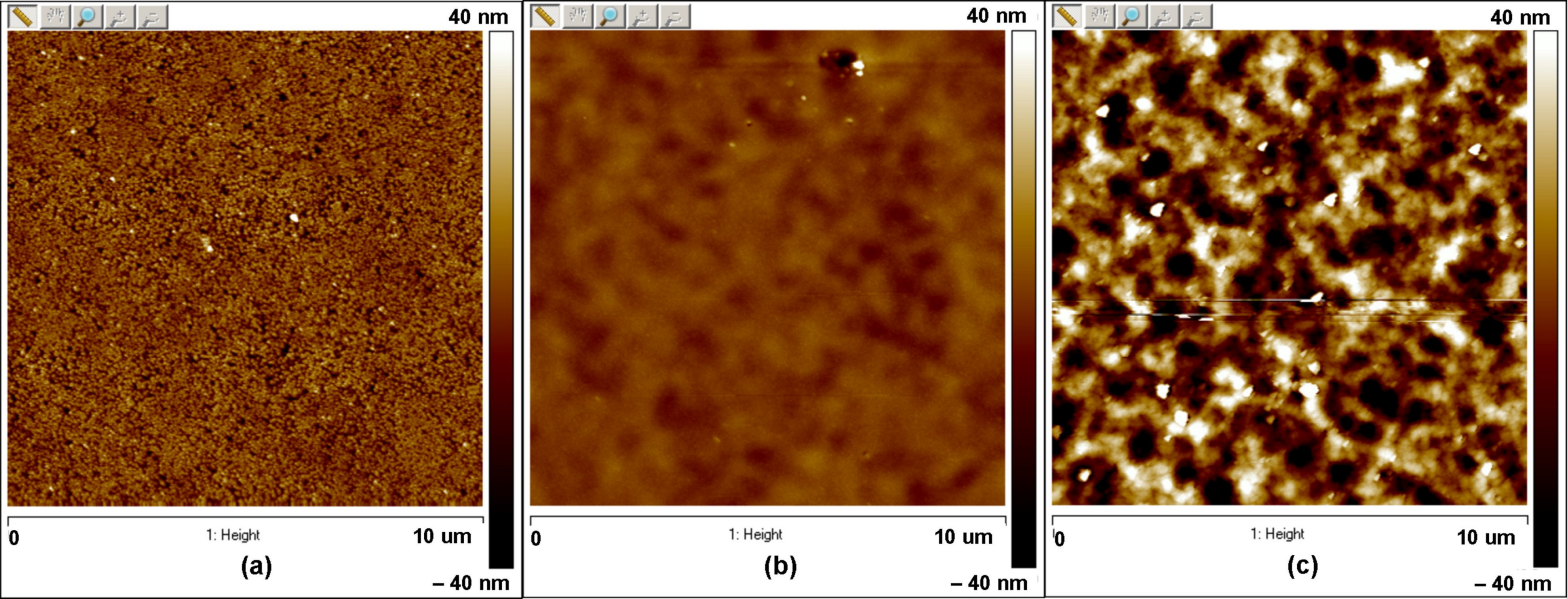
AFM images of the Si nanopowder surface before (a) and after irradiation by the cluster beam at an ion dose of 5.8 × 10^15^ cluster/cm^2^ and energy of 34.5 keV (b) and 17.3 keV (c).

This process is proved by comparison of AFM images before and after irradiation (Figure 5). On the surface before irradiation, individual nanoparticles can be observed (Figure 5a), whereas after 34.5 and 17.3 keV irradiation (Figure 5b and 5c) the particles are no longer visible, and instead, large (larger than the nanoparticle size of 60 nm) morphological structures are presented. Therefore, it can be suggested that an effective milling of the top layer silicon particles occurs. The space between the nanoparticles are filled by debris, which due to the limited lateral resolution cannot be observed. Such filling of the voids results in densification of the top surface layer. Therefore, as the number of contacts between neighbor nanoparticles increases, consequently, we can expect the weakening of the finite size effect. It means that the sputtering yield decreases and even reaches that of the bulk Si for 34.5 keV irradiation (Figure 3b). Thus, the decrease of the sputtering yield with the dose demonstrates continued weakening of the finite size effect due to accumulation of the debris in the top layer.

Bombardment at 17.3 keV revealed more effective sputtering (Figure 3b), accompanied by the roughening effect (Figure 4), which is related to the formation of the large morphological structures (Figure 5c). Higher sputtering can be explained by less effective formation of small Si particle debris on the surface due to lower projectile energy. Therefore, only a slight densification of the surface layer and retention of the finite size effect at given doses occurs, and an increased sputtering yield is observed even at lower energy. Nevertheless, at higher doses, the sputtering yield tends to decrease (Figure 3b), which suggest a slow accumulation of the debris, but slower than in case of 34.5 keV energy. The roughening effect is scarcely responsible for the increase of the sputtering yield, since the curvature of the appearing morphological structures is much larger than that that discussed in [6–7], ≈1 µm and 60 nm, respectively. Moreover, the roughening effect itself can be a consequence of the high sputtering yield. A more accurate explanation of the sputtering yield increase at the low energy requires additional study of the structure of the top layer.

It should be noted that the sputtering yields in Figure 3b were discussed without any assumption of the surficial densification. However, it can have an influence on the calculation of the number of sputtered atoms, which has been determined in supposition of the constant average density. The thickness of the dense surface layer can be estimated by characteristic sizes of the crater formation on the surface during cluster bombardment, which is considered to be the chief mechanism of the smoothing and sputtering processes of the bulk material at the high cluster energy. We propose that the effective debris formation and mixing of the material at the surface, i.e., the formation of the dense layer, occurs only in the finite layer with the thickness, which is equal to the crater depth. The crater depth in the case of the nanopowder target was found to be 3–5 nm at the same accelerating voltage [28]. Such a value is almost one order of magnitude lower than the sputtering depths in this study. Therefore, the influence of the surficial densification on the sputtering yield calculation is not significant.

**Figure 6:**
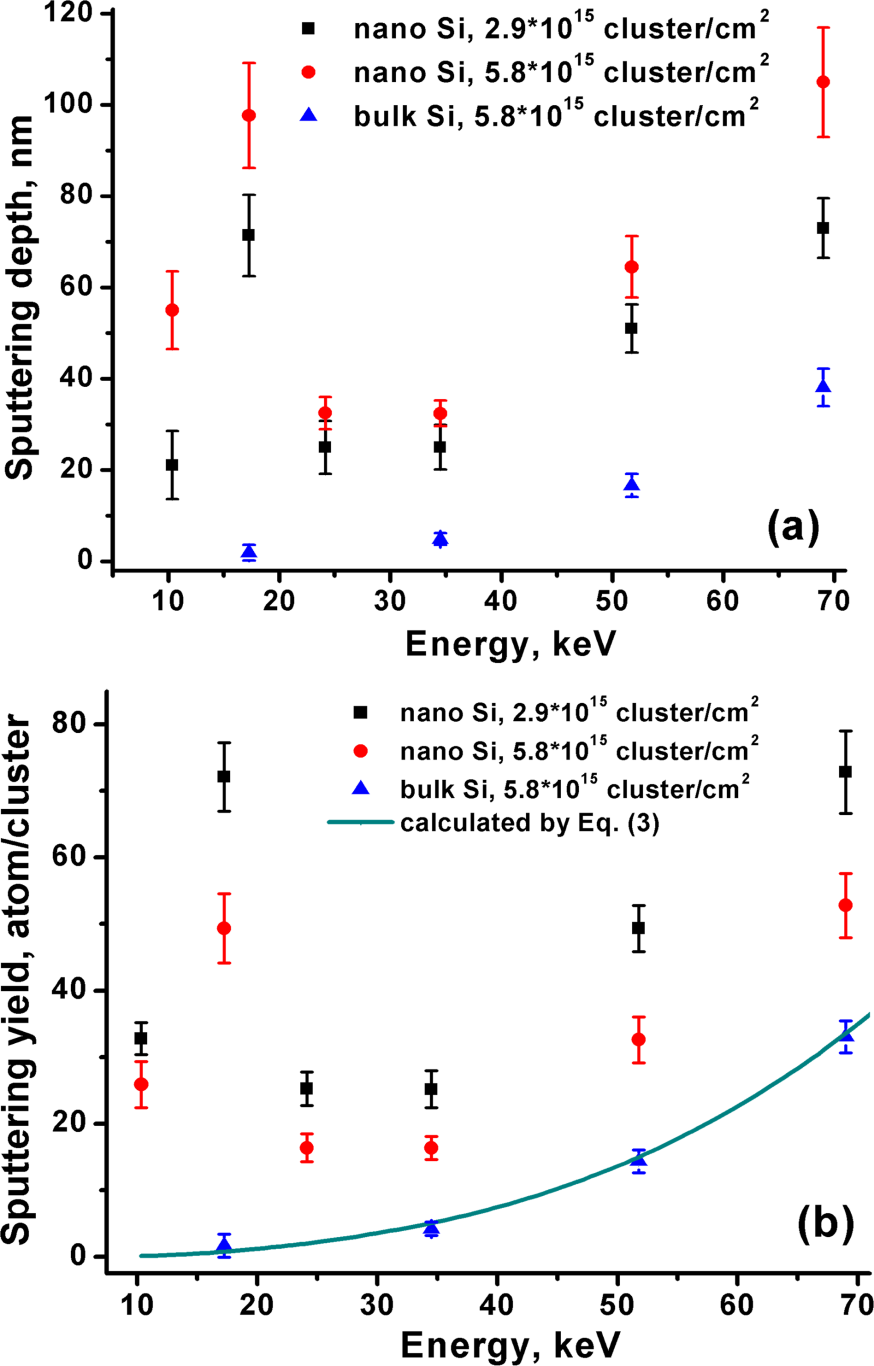
The sputtering depth (a) and sputtering yield (b) dependence of the Si nanopowder and bulk Si on energy at different ion doses. The sputtering yield of the bulk Si is fitted to the curve described by Equation 3.

Cluster energy dependence of the sputtering depth and sputtering yield are presented in Figure 6. Both characteristics in case of the bulk Si show continuous increase along with energy. The energy dependence of the sputtering yield of the bulk Si has already been studied in [15], where the sputtering yield was described by the power function:

[1] $$Y\propto {\left(k^{n}E-E_{\text{th}}\right)}^{1.5},$$

where *k* and *n* are parameters describing the collision of the clusters with the residual gas, *E* is the cluster energy, and *E*_th_ is the sputtering threshold energy. The threshold energy has been estimated to be about 6 keV. The same experimental data has also been fitted by the power function without incorporation of the threshold energy [34]:

[2] $$Y=\frac{N{\left[E/\left(AN\right)\right]}^{q}}{1+{\left[E/\left(AN\right)\right]}^{q-1}}\;,$$

where *N* is the number of atoms in the cluster, *A* = 57 eV and *q* = 2.25 are the fitting parameters. At low energy, the denominator is close to 1 and the equation is simplified to

[3] $$Y=N{\left[E/\left(AN\right)\right]}^{q}\;.$$

The functional dependence in Equation 1 and Equation 3 is the same except for the presence of the threshold energy *E*_th_ in Equation 1. In general, the existence of the threshold energy in the cluster collision with a solid-state surface seems rather unlikely, since such a collision is a collective interaction process of many atoms of the cluster with many atoms of the target and utilization of the threshold energy, which describes the binary collisions, can be unjustified. However, experimentally it is quite difficult to reveal the presence or absence of the energy threshold due to the exponential dependence of the sputtering yield on energy, i.e., a very small sputtering depth at low energy. Suggesting an absence of the threshold energy, we fit the experimental data to Equation 3 (Figure 6b) with the parameters *N* = 2900, *A* = 110 eV, and *q* = 2.95, which are comparable with those used in [34].

The sputtering depth and sputtering yield of the nanopowder samples do not show a simple increasing behavior (Figure 6). Significant sputtering of 33 atoms/cluster is observed even at 10.4 keV, whereas the sputtering of the bulk Si is not observed at this energy at the level of sensitivity. At 17.3 keV, unusual strong maxima are revealed and at an energy higher than 34.5 keV a continuous increase is observed. With increasing dose from 2.9 × 10^15^ to 5.8 × 10^15^ cluster/cm^2^, the sputtering depth does not increase linearly (Figure 6a), which is in agreement with the sublinearity of the dose dependence in Figure 3a. The sputtering yields decrease with increasing dose at all energies (Figure 6b), in agreement with the data presented in Figure 3b.

The sputtering yield of the nanopowder irradiated at 17.3 keV and 2.9 × 10^15^ cluster/cm^2^ is higher than that of the bulk Si by more than forty times, whereas when irradiated at 69 keV it is higher only by 2.2 times. With an increasing in energy up to 17.3 keV, the sputtering of the nanopowder reaches the maximum and occurs twice as much. Such unusual maximum in the energy dependence of the sputtering yield can be explained in terms of the above-developed concept based upon the competition of the finite size effect and debris formation. At low energy of 10.4–17.3 keV, the high sputtering yield of the nanopowder is caused by the finite size effect. An increase of the cluster energy in this region results in more effective sputtering due to an increase of the energy density in the impact zone and intensification of the finite size effect. At higher energy (17.3–30 keV), the sputtering yield decreases and reaches minimum. In this energy region, we propose the weakening of the finite size effect by the densification of the top layer due to particle debris formation by the energetic cluster bombardment. Then, at higher energy (30–69 keV), the sputtering yield again starts to increase, but here the increase occurs due to the higher energy delivered by the cluster ion to the surface, as it takes place during the sputtering of the bulk material (see the bulk Si sputtering (Figure 6b)).

**Figure 7:**
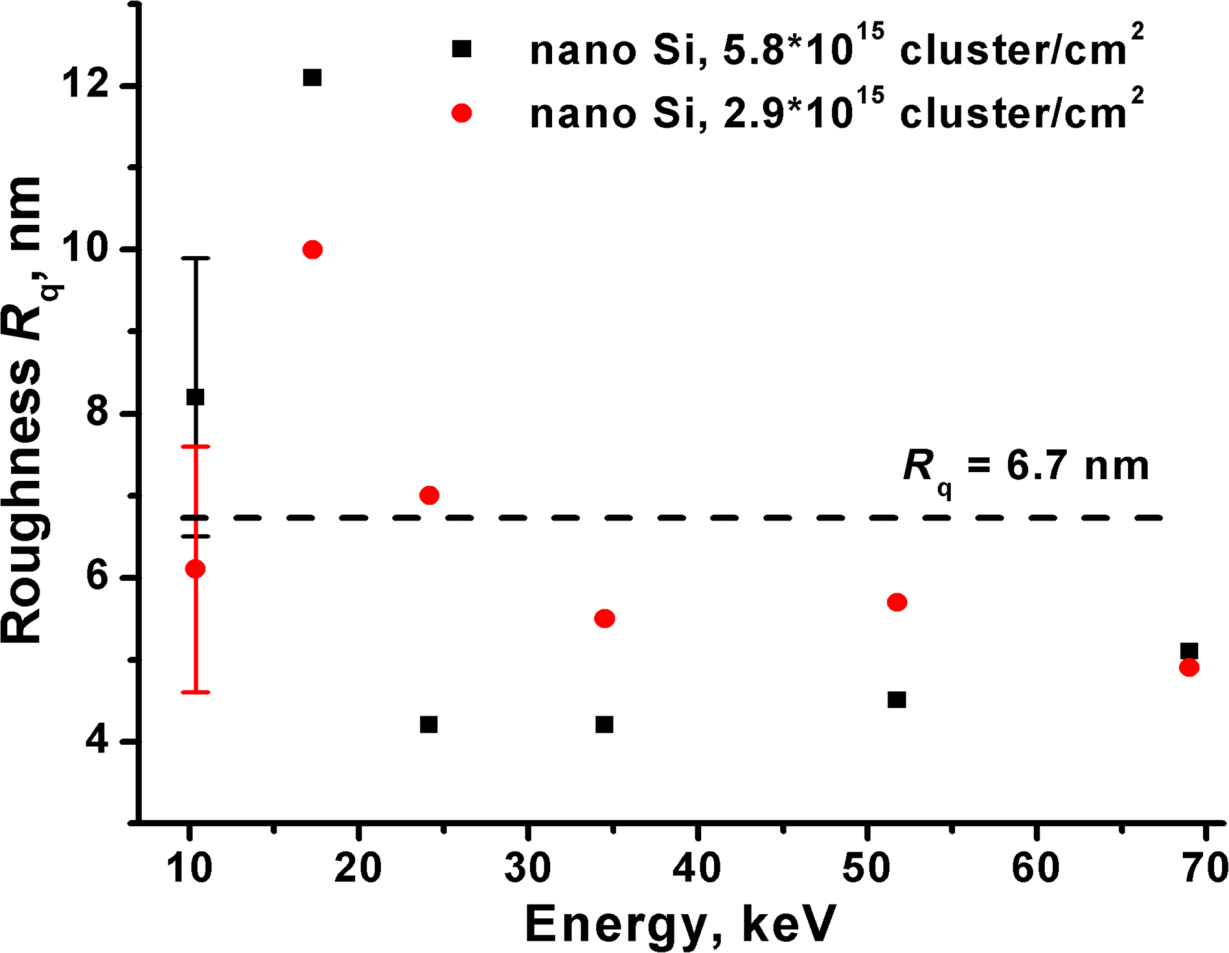
The roughness of the nanopowder Si versus the energy at different ion doses. The broken line represents the initial roughness.

Figure 7 shows the dependence of the roughness on the cluster energy. At low cluster energies (10–35 keV) the dependence demonstrates similarity with the energy dependence of the sputtering yield, i.e., the high sputtering yield is accompanied by the roughening effect. As mentioned above, the roughening effect is unlikely responsible for the sputtering increase and its mechanism as well as mechanism of the high sputtering yield are not clear yet.

At the energy of 17.3–34.5 keV the smoothing effect accompanied with the effective ion milling is appeared, which reduces the finite size effect and the sputtering rate. Further, at higher energy (up to 69 keV) the smoothing effect is retained. The dose dependence of the roughness due to a large experimental uncertainty cannot be revealed.

## Conclusion

In this work, we reported the results of the sputtering of silicon nanopowder (60 nm-sized particles) by Ar_2900_ clusters with energy of 10.4–69 keV. The samples were prepared as pressed tablets and irradiated at a right angle to the plane of the surface with a dose ranging from 7.2 × 10^14^–2.3 × 10^16^ cluster/cm^2^ at room temperature. The influence of the target density and cluster beam parameters (energy and dose) on the sputtering depth and sputtering yield were investigated. It was found that the sputtering yield exhibited an increase with decreasing average density of the pressed powder. The dose dependence of the sputtering yield of the nanopowder showed a decrease with increasing dose and the sputtering yield approached that of the bulk Si, which was dose independent. At doses lower than 2.9 × 10^15^ cluster/cm^2^ and energy of 17.3 keV, the sputtering yield was more than forty times higher as compared to the bulk Si. The energy dependence of the nanopowder sputtering yield exhibited unusually strong sputtering at 10.4–17.3 keV, whereas the sputtering yield of the bulk Si was negligible. In the moderate energy range (17.3–34.5 keV), the sputtering yield of the nanopowder decreased and then again increased at higher energies. A simple rational explaining the sputtering of the pressed nanopowder targets was developed. The increase of the nanopowder sputtering yield in comparison with the bulk sample was explained by the presence of the finite size effect. The observation of a smoother surface after cluster bombardment treatment suggested the densification of the top layer, which resulted in the weakening of the size effect, which, in turn, caused the decrease of the sputtering yield at high doses. The explanation of the sputtering yield energy dependence is based on the finite size effect strengthening the sputtering, on the one hand, and the process of nanoparticle debris formation, which weakens the finite size effect, on the other hand. The roughness measurement of the nanopowder samples after irradiation at 10.4–34.5 keV demonstrated a similar dependence with the sputtering yield. In this work, we studied a nanopowder with a fixed particle size of 60 nm in diameter. However, this is a key parameter considered in the finite size effect, and our further studies will aim to clarify the sputtering yield dependence on the particle size. The particular mechanism of the sputtering and the roughening effect will also be studied.
